# Report on the Physiology and Pathology of the Blood

**Published:** 1846-04

**Authors:** 


### PART IV.—PROGRESS OF MEDICAL SCIENCE.

### REPORT ON THE PHYSIOLOGY AND PATHOLOGY OF THE BLOOD.

### ARTICLE XI.

The following remarks upon the elaboration, composition, and relative constituent proportions of the blood; without professing to comprise a synopsis, even, of the most recently developed facts and opinions upon this subjec,t are presented to our readers for the purpose, merely, of calling their attention to the subject, and of showing the importance of making the very remarkable pathological changes that take place in this fluid, a means of diagnosticating and treating diseases.

It is a well known fact, that nutritious subtances taken as food, are, by the process of digestion, converted into a fluid substance called chyle, the composition of which is albumen and saline matters dissolved in water, with fat globules suspended in the fluid.

In its passage from the intestines, through the lacteals, to the mesenteric glands, and thence to the thoracic duct, the chyle undergoes many and important changes; the most remarkable of which is the conversion of its albumen into another organic principle called fibrine. "Albumen, then, may be regarded as the first proximate element; at the expense of which in conjunction with fatty matter all the tissues of the animal body are ultimately formed. Still this organic principle, as long as it remains in a state regarded by chemists as characteristic of it; exhibits no tendency to become organized, and it is only when it has been subjected to certain peculiar vital influences, and, perhaps, undergone a change in its chemical constitution, or, in other words, has been converted into fibrine, that any such tendency manifests itself.

As the consequence of this change, the fibrine increases, and the albumen lessens, whilst, at the same time, the oil-globules undergo an evident diminution. It is not conceivable that the fibrine should be at once formed from the oil-globules, since albumen is much more ready to undergo organization and vitalization, and is always the preceding principle to fibrine. The fibrine must, therefore, be produced at the expense of the albumen, whilst new albumen is elaborated from the oily matter.

The following table presents a view of the relative proportions of the three principle ingredients in the chyle in different parts of the absorbent system, and gives an idea of its advance in the progress of assimulation.

| | |
|---|---|
| In lacteals, from intestines, to mesenteric glands. | Fat in maximum quantity.<br>Albumen in minimum quantity.<br>Fibrine almost entirely wanting. |

| | |
|---|---|
| In lacteals, from mesenteric glands to thoracic duct. | Fat in medium quantity.<br>Albumen in maximum quantity.<br>Fibrine in medium quantity. |
| In thoracic duct. | Fat in minimum quantity.<br>Albumen in medium quantity.<br>Fibrine in maximum quantity. |

The fluid, drawn from the thoracic duct is frequently observed to present a decided red tinge, which increases on exposure to air. This tinge is due to the presence of true blood corpuscles, smaller, however, than those of the true sanguineous fluid.

As to the composition of the blood then, it may be said to be composed of water holding albumen, fibrine and saline matters in solution, having particles of a red colour floating in the fluid. These, the true blood-globules, are flattened discs, having a circular outline, and composed of three parts, the capsule, the nucleus, and the contained matter, thus constituting a complete nucleated cell. It is a peculiarity in the composition of these globules that they each contain a small proportion of iron: the red colour of the blood is due to their presence.

Blood drawn from the body, and left to itself, soon presents a new arrangement of its organic elements. The fibrine coagulates and separates from the fluid portion, attracting and enclosing in its meshes the red globules, thus forming what is called the coagulum or clot. The remaining portion, designated serum, is composed of water holding in solution the albumen and saline matters.

According to M. Andral, the usual or normal proportions of these different ingredients in the blood, are as follows:

| | | |
|---|---|---|
| Of each element in 1000 parts, | Fibrine, | 3 |
| | Globules, | 127 |
| | Solid matter, dissolved in serum, | 80 |
| | Water, | 790 |

The proportion of fibrine may vary in health from 2.5 to 3.5, that of the globules from 110 to 140. The clot formed by the coagulation of blood, being dependant for firmness upon the fibrine, and for colour and volume upon the amount of globules, indicates, by its density and more perfect reticulated structure, an exuberance of fibrine, and by its softness, bulk and high colour, a comparatively large proportion of globules.

Blood coagulated slowly, and in which the proportion of fibrine is large, as compared with the globules, permits the colouring matter to settle to the bottom of the clot, thus leaving a nearly colourless layer of fibrine upon its surface, long since known to physicians and described by writers as the *buffy coat.* In cases where the proportion of fibrine is large, and the coagulum firm, it presents a concave or cupped surface.

All the organs of the body being dependant upon the blood for nutrition and support, are, consequently, deranged in their functions and subject to change of structure whenever this fluid becomes deteriorated in quality, or changed as to the relative normal quantities of its constituent proportions.

Among the most important and easily detected pathological changes which take place in the blood, are those resulting from a want of the due proportions of its elements, such as an exuberance or deficiency of globules, increased or diminished amount of fibrine, want of albumen, saline matters, &c.

Of the abnormal conditions and diseases of the system, in which such changes in the constituent proportions of the blood are most apparent, may be enumerated Plethora, Anæmia, Pyrexiæ, Phelgmasiæ, Hemorrhages and Dropsies.

Contrary to the generally received opinion that the peculiar condition of the system in plethoric individuals depends upon exuberance of blood, it appears from the researches of Andral, that the marked peculiarity of this fluid in the above named condition of the system, is an increase in the amount of its globules. In 31 bleedings of plethoric subjects, in each of which an analysis of the blood was made by this distinguished pathologist, the average amount of globules in 1000 parts was 141, the normal quantity being 127. Hence it is that the blood of such persons is high colored, presenting, after coagulation, a comparatively small amount of serum, a voluminous clot filled with fluid, and soft for want of fibrine, but never incrusted with the buffy coat.

Coincident with an excess of globules in the blood of the plethoric, there are certain physiological and pathological phenomena dependant, as it seems, upon this peculiar condition of the fluid. All the functions of life are more active, digestion quick, respiration full, circulation rapid, with a degree of general excitability of the whole organism; thus showing that the blood of plethoric individuals is highly exciting, and of a stimulating character. This truth is made more apparent also, by the fact, that such persons are subject to vertigo, dizziness, ringing in the ears, &c., symptoms produced, without doubt, by the passage of an excessive number of globules through the brain. Plethoric persons are, also, more liable than others to active hemorrhages and apoplectic effusions, the result, evidently, of the absence of the due proportion of coagulable matter in the blood.

By blood-letting, abstemiousness, and general antiphlogistic treatment, the number of blood-globules are readily diminished.

It appears, then, that an excess of the globular element of the blood is a constant distinguishing characteristic of plethora; a fact leading to the inference of that, which is in reality true, viz: that in anæmia,—a condition of the system directly opposite to that of plethora,—the amount of globules falls far below the natural standard.

This anæmic condition of the system, is characterised by paleness of countenance, general muscular debility, nervousness, neuralgia, vertigo, dispnœa and palpitations of the heart upon the slightest effort.

An example of spontaneous anæmia, in which most of the above named symptoms are always present, is that not uncommon but peculiar disease of females, chlorosis.

This want of the globular element in the blood is often the effect of debilitating diseases and profuse hemorrhages. Pregnant women, also, during the last months of gestation, often become anæmic. Andral observes, "I have found as the average of the proportion of globules in 16 cases of commenceing anæmia, the cipher 109, and in 24 cases of confirmed anæmia the cipher 65"—127 as above stated being the normal quantity.

The physical properties of the blood drawn from anæmic patients is perfectly characteristic of its condition. For want of globules the coagulum is small, and swims in a large amount of perfectly colourless serum; but, as there is no want of fibrine, it is firm and generally presents upon its surface a well marked buffy coat, resulting from the absence of globules in this upper stratum of the fibrinous mass.

Thus it is seen that the presence of the buffy coat is not always a diagnostic sign of inflammation, since it appears upon the blood of patients whose general condition, evidently, requires a course of treatment directly the opposite to that, generally considered to be most efficacious in the phlegmasiæ.

In order to increase the amount of blood globules and thus restore the blood to its normal condition, those means are to be employed which are generally believed to give tone and vigour to the system, such as nutritious food, fresh air, exercise and tonics, of which, the preparations of iron are most effectual.

As an example of the good effects of proper treatment in such diseases, Andral mentions the case of a young lady, who, in 3 months, had the amount of globules so much increased as to present a change in that short space of time, from a confirmed anæmic to a well marked plethoric condition.

The *pyrexiæ* are another class of diseases in which the change in the blood, if not so well marked and defined, are still of great practical importance as indications for diagnosis and treatment.

These affections being characterised like the phlegmasiæ, by general febrile excitement, an attempt has been made, by several distinguished writers, to make it appear that the fever attendant upon them is always symptomatic of local disease, and that they should all be classed together with simple inflammatory affections.

"Such pretensions," remarks Andral, "cannot be maintained. The pyrexiæ exist as diseases apart; the causes which often develop them, the symptoms which characterize them, the special nature of the alterations which they produce in the solids, are already enough of grave reasons for not confounding the pyrexiæ and the phlegmasiæ;" "but the analysis of the blood comes still more to establish a very remarkable difference between the one and the other class," for "in the phlegmasiæ there are always two constant alterations which march together, that of the solid and that of the blood, it is no longer the same in the pyrexiæ; in these diseases in reality, the only phenomenon which never fails, is the fever itself."

But although it is true as above stated, that the pyrexiæ may exist without any appreciable alteration in the solid or fluids, yet there never is in these, as in inflammatory diseases, an increase in the amount of fibrine. On the contrary, this organic element, even in the milder forms of these diseases, is generally diminished, and in well marked cases, it is not only uniformly lessened in quantity, but becomes deteriorated in quality, as appears from the imperfect structure of its coagulum.

Blood drawn from an individual suffering under the influence of typhus, typhoid, miasmatic fevers, or other forms of pyrexiæ, coagulates slowly. The fibrine, being small in quantity, and of an inferior quality, the clot is voluminous, imperfectly separated from the serum, soft and easily broken; in fine, the blood of such persons presents appearances indicative of low vitality, and a want of coagulable matter.

In the above described morbid condition of this fluid, there being a diminished amount of fibrine, but, at the same time, a normal quantity, or a relatively increased proportion of globules, the buffy coat, resulting, as before stated, either from an exuberance of the former, or a paucity of the later element, is never present.

This affords another important distinguishing characteristic between the phlegmasiæ and pyrexiæ. "I may affirm," says Andral, "that I have never met with it (the buff) unless there was phlegmasial complication, either in slight or severe typhoid fever, in measles, in scarlatina, or in variola."

As the result of a want of fibrine and a normal tendency to coagulation in the blood in the advanced stages of the adynamic or putrid type of the pyrexiæ, it has a tendency to exude from its vessels; hence the effusion of blood into the pustules in variola, the hemorrhages in malignant scarlatina, and the epistaxis and bleeding of the gums in the advanced stages of severe typhus and typhoid fevers.

The numerous sanguineous congestions in this class of diseases, mistaken often, for inflammations, are evidently the effects of the absence of a due proportion of coagulable matter in the blood. The enlargement and softening of many organs, as the spleen, are also, the effect, many times, of an accumulation of such blood in their structure.

As to the origin of the pyrexiæ, it may be said, that it is by no means probable that the want of fibrine is the primary cause of these diseases, for it seems incontestible, "that the specific cause which gives them birth, acts upon the blood in such a way, that it tends to destroy its spontaneously coagulable matter." "If this cause acts with slight energy, or if the economy resists it, the destruction of the fibrine is not accomplished; if, on the contrary, the cause continue to act with all its intensity, and the forces of the organism be in fault, the destruction of the fibrine will commence either at the very beginning of the disease, which is very rare, or a certain period after its commencement: all this applies itself equally well both to the typhoid fevers, and to the eruptive fevers."*

It is a fact well known to the profession, that there are but few diseases of the class pyrexiæ, for which we have remedies of known efficacy; and it is equally true, that of the means of restoring the blood, in these diseases, to its normal condition, we are equally uninformed. It has been suggested by some, that the administration of such agents as favour the coagulation of blood, such as acids, &c., might prove beneficial.

We now pass to the consideration of another, perhaps the most important class of diseases—the phlegmasiæ,—which, though characterized by general febrile excitement like the pyrexiæ, present at the same time, a directly opposite condition of the blood.

Meckel has defined inflammation to be "congestion with a tendency to new production." This definition is, probably, as nearly correct as any that can be given; for from the very beginning to the termination of every inflammatory affection, there is a constant tendency in the blood, in proportion to the extent and severity of the disease, to the production and increase of its fibrinous element. The augmentation of the quantity of fibrine is so constant a sign of inflammation, that if we find more than five parts in 1000 in the course of any disease, we may positively affirm that some local inflammation exists.

During the active stage of inflammatory diseases, blood drawn from the patient presents all the characteristics indicative of an increase in the amount of fibrine. The coagulum is small, but firm, and comparatively free from serum, presenting, in most cases, when properly drawn, a well marked buffy coat, with a concave or cupped surface. The buffy coat then; excepting in anæmic conditions of the blood, where the amount

*Andral.

of fibrine, not being actually increased, is large only as compared with the diminished proportion of globules; always indicates an increase in the fibrine, and consequently the existence of some inflammatory affection.

The condition of the system, at the time of an inflammatory attack, seems not to influence the consequent increase of fibrine. A person may be exhausted by chronic disease, or present a confirmed anæmic condition, yet, upon the accession of an inflammatory affection, there is in these, as in other cases, as constantly an increase of this constituent. In chlorotic patients, for instance, who have been attacked by acute articular rheumatism, bronchitis, pneumonia or erysipelas, the blood has been found to contain 6, 7, and even 8 parts of fibrine; and in cases of the pyrexiæ even, such as typhoid, typhus and miasmatic fevers, causing a diminution of fibrine, if, in the course of the disease, there supervenes an inflammatory affection, the amount of this element is always increased; thus presenting two forces acting upon the blood, one to diminish, the other to increase the proportion of this, its spontaneously coagulable element.

We may mention in this connection a curious fact, established by Gavarret, that the blood of animals, dying of hunger, shows an increase in the amount of fibrine, an effect, as appears from examination, of inflammation of the coats of the stomach.

As to the comparative increase of fibrine, in inflammation of different tissues, it may be remarked, that of all diseases, pneumonia and acute articular rheumatism, are those in which the augmentation of this element is the greatest; the amount of fibrine in these affections often rising to the proportion of 10 in 1000.

In slight inflammation of the mucous membranes, unattended by febrile excitement, there is little or no appreciable change in the blood; but whenever the local disease becomes sufficiently intense to produce general reaction, the proportion of fibrine is found simultaneously to increase. It may in truth be stated, as a general fact, that independant of the organ affected, the accession and continuance of the accompanying fever bears a close relation to the constantly progressive changes in the sanguineous fluid. It seems not unreasonable to suppose, then, that the blood, circulating as it does through every organ and tissue of the body, thus surcharged with an undue proportion of its fibrinous element, produces that morbidly excited condition of every part, termed sympathetic fever.

The blood of individuals affected with mercurial ptyalism, presents always an increase of its fibrine, in proportion to the extent and severity of the inflammation. To illustrate, we give the following brief synopsis of three cases recorded by

Andral. In the first case, profuse salivation was produced by 18.5 grs. calomel, augmenting the proportion of fibrine in the blood to 4.5. In the second case 9.5 grs. of calomel gave rise to very acute inflammatory action, increasing the fibrine to 5; and in the 3d case, more violent inflammation was produced than in either of the preceding, by the combined internal and external use of mercurials, raising the proportion of fibrine to 8.4.

Thus we see, that inflammation produced by mercury, appears not to differ in its effects from that produced by other causes. This fact may lead some to doubt, with good reason, the propriety of producing salivation in acute inflammatory diseases, and thus adding to the amount of fibrine already too abundant. It seems not, however, to contraindicate the more moderate use of the mineral, for it appears that the inflammation, not the medicine, caused the morbid change.

On the other hand, there seems to be some slight indication in the above fact, for the production of ptyalism to restore fibrine, and thus supply the deficiency in the pyrexiæ, such as typhus and typhoid fever.

Mercury is a remedy which has been, and is still used and recommended by practitioners, empirically, as it would seem, unless the above is a good reason for its use. Let us not, however, form hasty conclusions upon this point. In our contentions with disease, plausible reasons may justify the trial of a remedy, but favorable effects only recommend its general use.

Inflammations of the skin, from severe burns, erysipelas, blisters, &c., give rise also, to augmentation of fibrine in the blood. This fact suggests the probability, that the consequent increase of this element in cutaneous inflammatory affections, may be the principle and perhaps only reason, why blisters and other severe external irritants increase the sympathetic fever, and thus act unfavorably in the acute stages of these diseases. It may be true also, that the well known good effects of these remedies, in fevers of a typhoid type, and in old chronic affections, are, in part, the immediate results of an increase of fibrine, consequent upon the inflammatory action thus artificially produced.

It seems then, that inflammation, whatever may be the causes and circumstances attending its accession and continuance, appears and advances in most cases, simultaneously with the production and increase of this constituent of the circulating fluid.

As an exception, however, to the general rule, that increase of fibrine is accompanied with inflammatory action, it may be remarked, that pregnant women, during the last months of gestation, present blood, containing this element in a much higher proportion than the normal standard, varying for instance, from 4 to 5 in 1000 parts. It seems then, that "the blood on these occasions shows a remarkable tendency to assume the character of inflammatory blood; and assuredly there is matter for reflection in the relation which may exist between the modification then affected in the blood, and the development of those peculiar attacks, generally of an inflammatory aspect, to which women in child bed are so liable."

As to the treatment of inflammation, it may be remarked, that the condition of the blood in these diseases seems to indicate the employment of such means as tend most directly to diminish the amount of its fibrinous element. Unfortunately however, no agent is known to the profession by means of which we can produce this effect promptly, and to such an extent as to give us unmistakable evidence for or against this apparently legitimate conclusion.

Venesection has been found, by experience, to be one of the most effectual means of moderating and checking the progress of the phlegmasiæ. The most obvious effect of blood-letting, is that of abstracting a comparatively large proportion of the blood's most stimulating element, the globules. It acts favorably also by lessening, in common with the other ingredients, the exuberant fibrine, and thus removing, to some extent, the apparent cause of febrile action; and lastly, by diminishing the amount of blood circulating and thus favoring the absorption of fluids, it causes a dilution, and consequently, a more general diffusion of all its constituents. Cathartics, low diet, and most others of the class of antiphlogistic remedies, produce their beneficial effects, very probably, by diluting and diminishing the active constituents of the blood, and thus rendering this fluid less stimulating to the system generally, and to inflamed organs in particular.

Since the establishment of the important truth, that excess of coagulable matter is a distinguishing characteristic of inflammatory blood, it is said that well marked favorable results have been obtained from the administration of remedies, such as alkalies and other chemical agents, the well known effect of which, is to retard the coagulation of blood, and, as is believed by many, to prevent the rapid formation of fibrine. Nitrate of potash, for example, is a well known effectual remedy in this class of diseases, which, as it prevents the coagulation of blood after it is drawn, and even in the vessels, when injected into the veins, may depend, in part, for its beneficial effects in disease, upon this property.

In concluding these remarks upon the phenomena cöincident with a pathological condition of the circulating fluid with regard to the fibrine, it may be stated, that among the most important of these, is a spontaneous tendency to loss of blood—the concomitant always to a deficiency of the coagulable as compared with its globular element.

It appears from facts before stated, that this want of normal proportions between these constituents is present in two apparently opposite conditions of the system—the plethoric and typhoid—in each of which, the blood presents the peculiar characteristics of excess of globules in the former, and deficiency of fibrine in the latter condition. Hence it is, that hemorrhage may be either active or passive, according as it occurs in one or the other of these abnormal conditions of the circulating fluid.

In plethoric persons, bleeding at the nose and other hemorrhages, if not profuse, or in such locations as to interfere with the vital functions, often relieve the unpleasant symptoms to which, as before stated, these individuals are sometimes subject. This is because a bleeding or hemorrhage, if not carried beyond a certain point or excessive, diminishes the globules proportionally more than the fibrine, and thus for a time, removes the plethoric condition; but if, on the other hand, the abstraction of blood be carried too far, or if the active hemorrhage continue long and profuse, the fibrine begins to diminish at first, to the same, and afterwards, to a greater extent even than the globules, thus presenting at length, a condition of the blood, in which there is a deficiency of fibrine as compared with the globules, and thus changing its condition from that productive of a comparatively harmless escape of excess of globules, to that coincident with the more obstinate and dangerous disease, a passive hemorrhage.

From remarks, which have preceded upon this subject, it appears that the condition of the blood, favorable to passive hemorrhage, is present in the advanced stages of all the most malignant pyrexiæ, and as an effect of this morbid condition we have in these diseases, effusion of blood into the tissue of organs and under the cuticle, producing engorgement and petechial spots and patches; hemorrhages both internal and external; collections of blood in the cavities, and frequent bleedings from the gums, nose, rectum and other parts.

Thus we see that a loss of the fibrine of the blood is one of the most disastrous in its effects of all its pathological changes, a truth calling loudly upon the profession for some means of preventing the destruction of this important element.

No remedy is known, however, of sufficient activity and promptness in its effects, to modify this condition of the blood, to any appreciable extent, in the most malignant forms of this class of affections. It is evident then, that our attention, for the present, should be directed to the first stages, and milder forms of these diseases, and to the nature and mode of action of those remedies which have been found most effectual in these more simple cases, where their effects can be seen and appreciated.

In all of the more chronic and milder forms of this class of diseases, with blood deficient in fibrine, such as scurvy, &c., more or less, of the following symptoms, always present themselves: pale, livid and dusky countenance, petechiæ, ecchymoses, soft, swollen and bleeding gums, debility, general numbness, heaviness of the head and vertigo. Blood drawn from an individual with such symptoms, coagulates slowly and imperfectly, the clot is, generally, large but soft, and often in separate fragments, never however presenting the buffy coat.

As to the therapeutic agents to be employed in diseases of this class, it may be remarked, that the causes, giving rise to them, as well as certain attendant conditions of the blood, give us some important indications.

The causes which most commonly give rise to this abnormal condition of the blood, are a damp and vitiated atmosphere, insufficient and improper food, such as that deficient in albumen and fibrine, the excessive use in food or otherwise of neutral salts or alkaline substances, such as common salt, salæratus, &c. Of the bad effects of the last named class of chemical substances, we have ample evidence in the condition of the blood coincident with a loss of fibrine.

It is true, that the loss of a large proportion of the blood's coagulable element in these diseases, is the change made most appreciable by chemical analysis. "But is this the ultimate alteration we are permitted to arrive at? Before the fibrine decreases, has there not been some other change of composition in the blood, of which the depression of fibrine below its normal average is itself only the consequence? Upon this subject some facts may be cited. It may be remarked, that on throwing into the veins of living animals, a concentrated solution of subcarbonate of soda, an almost fluid blood was found in the bodies of these animals when dead, and that during life, their symptoms were analogous to those observed in diseases, in which the older writers admitted a state of dissolution of the blood. It may also be remarked that some authors declare, that they have found an excess of alkaline matters in the imperfectly coagulated blood of persons who died of low fevers or scurvy."* It is also true that blood drawn from patients with scurvy and other diseases, depending upon the same cause has presented the same characteristic.

It would appear then, that the treatment indicated in such cases, is to counteract the influence of the above named causes, by introducing in their place, an opposite train of influences, such as exercise, uncontaminated air, a plentiful supply of nutritious, nitroginized food, and the displacement of alkaline ingesta by acid substances.

That the acids act favourably in these diseases, by neutralizing the excess of alkaline constituents in the blood, and favouring its coagulable tendency, is a view of their modus operandi

* Andral.

of recent date. Whatever may be their mode of action, however, there can be no doubt with regard to their utility, or of the good effects of the general treatment above described, numerous results and long experience having established its efficiency.

In conclusion, it may be remarked that deficiency of albumen, is another very important alteration, not yet referred to, in the relative proportions of the blood's constituents, concomitant, always, with a tendency to dropsical effusions. Want of space however, compels us to defer, for the present, a consideration of this part of the subject. We hope, however, that the preceding partial exposition of some of the more modern, but generally adopted views, with regard to the composition and different conditions of the blood, imperfect as it is, and too limited for an exposition of the relation of facts referred to, may serve to direct attention to the importance of the subject and to the interesting facts and conclusions, which several distinguished men, now engaged in its investigation, are, from time to time, communicating to the profession.

